# Pleural and Thoracic Air-Leak Complications After Hematopoietic Cell Transplantation: Diagnosis and Procedural Management

**DOI:** 10.3390/arm94050071

**Published:** 2026-10-01

**Authors:** Aryan Shiari, Lamia Aljundi, Ayman O. Soubani

**Affiliations:** 1 Department of Pulmonary and Critical Care Medicine, Mayo Clinic Health System, 1221 Whipple St., Eau Claire, WI 54703, USA; 2 Division of Pulmonary, Critical Care, Hyperbaric, Allergy, and Sleep Medicine, Department of Medicine, Loma Linda University School of Medicine, Loma Linda, CA 92350, USA; 3 Division of Pulmonary, Critical Care, and Sleep Medicine, Wayne State University School of Medicine, Detroit, MI 48201, USA

**Keywords:** hematopoietic cell transplantation, allogeneic transplantation, pleural effusion, thoracentesis, chronic graft-versus-host disease, serositis, thoracic air-leak syndrome, chylothorax, pleural infection, procedural safety

## Abstract

**Highlights:**

**What are the main findings?**

- Direct evidence on pleural and thoracic air-leak complications after HCT remains limited and is concentrated in adult allogeneic recipients.
- Timing, imaging pattern, and selective pleural fluid analysis help distinguish infection, volume overload, cGVHD serositis, chylothorax, and structural air-leak disease.

**What are the implications of the main findings?**

- Pleural interventions after HCT should use ultrasound or image guidance and procedure-specific rather than uniform hemostatic thresholds.
- Standardized HCT-specific reporting is needed to define procedural safety, management thresholds, and outcomes.

**Abstract:**

Pleural and thoracic air-leak complications after hematopoietic cell transplantation (HCT) are clinically important but underrepresented in broad post-transplant pulmonary reviews. Direct evidence is concentrated in adult allogeneic HCT. In a cohort of 618 adults, clinically significant pleural effusion had a 1-year cumulative incidence of 9.9% (95% confidence interval, 7.7–12.5%). A separate computed tomography-based cohort of 178 first allogeneic HCT recipients reported a day-100 cumulative incidence of 41.0%; these estimates are not directly comparable because the populations, ascertainment methods, endpoints, and follow-up differed. In 50 HCT recipients undergoing thoracentesis, a specific diagnosis was established in 26%, with 5 pneumothoraces and 0 hemothoraces. Thoracic air-leak syndrome is uncommon but carries poor outcomes in reported adult and pediatric series. This focused narrative review distinguishes direct HCT evidence from data extrapolated from hematologic malignancy, general pleural practice, critical care, interventional radiology, and cellular therapy. We organize evaluation by urgency, transplant phase, and radiographic pattern; define chronic graft-versus-host disease serositis as a diagnosis of exclusion; summarize chylothorax and pleural infection management; and provide procedure-specific safety considerations for thoracentesis, small-bore drainage, indwelling pleural catheters, intrapleural enzyme therapy, and pleural biopsy. No prospective HCT-specific trials validate these procedural pathways. Clinicians should therefore use ultrasound or image guidance; apply procedure-specific rather than uniform hemostatic thresholds; and integrate infection risk, platelet trajectory, anticoagulant exposure, oxygenation, operator setting, and expected benefit. The two clinical frameworks presented are proposed and non-validated.

## 1. Introduction

This review provides an evidence-calibrated approach to diagnosing and managing pleural effusions and thoracic air-leak complications after hematopoietic cell transplantation (HCT). Its objectives are to distinguish direct HCT evidence from extrapolation, organize evaluation by transplant phase and radiographic pattern, and provide procedure-specific guidance for pleural interventions.

Pleural complications matter because they may signal infection, fluid overload, organ dysfunction, malignancy, drug toxicity, chronic graft-versus-host disease (cGVHD), or structural lung injury and may prompt invasive procedures in cytopenic, immunocompromised patients. Broad HCT pulmonary reviews appropriately emphasize pneumonia, idiopathic pneumonia syndrome, diffuse alveolar hemorrhage, organizing pneumonia, bronchiolitis obliterans syndrome (BOS), and other parenchymal or airway toxicities; dedicated pleural guidance remains fragmented [1,2,3].

The contemporary epidemiology also depends on ascertainment. A clinically based adult allogeneic HCT cohort reported a 1-year cumulative incidence of 9.9%, whereas a separate computed tomography-based cohort reported a day-100 cumulative incidence of 41.0% [4,5]. These estimates answer different questions and should not be combined.

The review focuses on pleural fluid and pleural air. Thoracic air-leak syndrome (TALS) is included because pneumothorax requires pleural decision-making, although pneumomediastinum, pneumopericardium, subcutaneous emphysema, and pulmonary interstitial emphysema are not themselves pleural disorders. Chimeric antigen receptor T-cell (CAR-T) therapy is not HCT and is discussed only as adjacent evidence relevant to differential diagnosis in programs that deliver both therapies.

## 2. Literature Search and Evidence Framework

This focused narrative review searched PubMed/MEDLINE, Google Scholar, and reference lists through 15 September 2026. The core PubMed search was (“hematopoietic cell transplantation”[Title/Abstract] OR “hematopoietic stem cell transplantation”[Title/Abstract] OR “bone marrow transplantation”[Title/Abstract]) AND (“pleural effusion”[Title/Abstract] OR “thoracentesis”[Title/Abstract] OR “serositis”[Title/Abstract] OR “pneumothorax”[Title/Abstract] OR “pneumomediastinum”[Title/Abstract] OR “chylothorax”[Title/Abstract] OR “pleural infection”[Title/Abstract] OR “indwelling pleural catheter”[Title/Abstract]).

A second procedural search was (“thoracentesis”[Title/Abstract] OR “chest tube”[Title/Abstract] OR “indwelling pleural catheter”[Title/Abstract] OR pleuroscopy[Title/Abstract] OR “tPA DNase”[Title/Abstract]) AND (“thrombocytopenia”[Title/Abstract] OR “coagulopathy”[Title/Abstract] OR “hematologic malignancy”[Title/Abstract] OR “immunocompromised”[Title/Abstract]). Additional searches combined cGVHD, thoracic air-leak syndrome, pleuroparenchymal fibroelastosis, dasatinib, CAR-T, lymphangiography, and thoracic duct embolization with the relevant pleural term.

Eligible sources were English-language human studies that reported pleural effusion, pleural air, serositis, chylothorax, pleural infection, or pleural procedure outcomes in adult or pediatric HCT recipients. Cohorts, case–control studies, case series, case reports for rare phenotypes, guidelines, consensus statements, and systematic reviews were included. Evidence from hematologic malignancy, critical care, interventional radiology, general pleural populations, and CAR-T therapy was included only when direct HCT evidence was absent or when it informed differential diagnosis. Animal studies, in vitro studies, and broad HCT pulmonary reviews without pleural or air-leak data were excluded from the evidence tables.

A.S. performed the searches and initial source selection; all authors reviewed the included evidence and the final interpretation. We did not preregister a protocol, perform duplicate screening, use a formal risk-of-bias instrument, or conduct meta-analysis. Accordingly, this work is a narrative clinical review rather than a systematic review. We label evidence as direct HCT evidence or extrapolated evidence and report study design rather than assigning an unvalidated quality grade [6].

## 3. Epidemiology and Timing-Based Diagnosis

### 3.1. Direct Post-HCT Estimates

Direct epidemiologic evidence applies predominantly to adults undergoing allogeneic HCT. Modi et al. retrospectively evaluated 618 adult allogeneic HCT recipients and identified 71 clinically significant pleural effusions. The cumulative incidence was 9.9% (95% confidence interval [CI], 7.7–12.5%) at 1 year and 11.8% (95% CI, 9.4–14.6%) at 5 years; median onset was day 40. The endpoint captured clinically recognized events rather than systematic detection of small asymptomatic fluid collections. Early events clustered within 100 days and were commonly attributed to infection or volume overload; later events were more often associated with cGVHD serositis or organizing pneumonia-associated effusion [4].

Masuda et al. used serial computed tomography review in 178 first allogeneic HCT recipients and identified new pleural effusions in 106 patients, with a day-100 cumulative incidence of 41.0% and median onset at 38 days. Two-year overall survival was 86.1%, 60.0%, 59.6%, and 18.8% among patients with fluid in zero, one, two, and three body cavities by day 100, respectively [5]. The 9.9% and 41.0% estimates are not directly comparable because the cohorts, detection methods, clinical thresholds, endpoints, and follow-up periods differed. Together, they show that radiographic fluid is common, whereas clinically actionable effusion is less frequent.

Pediatric, autologous HCT, and modern haploidentical HCT data are much thinner, and no study provides a reliable pooled incidence across transplant platforms. Pleural effusion should therefore be interpreted as a phenotype and risk marker, not as a single-cause complication with one generalizable frequency.

### 3.2. Pre-HCT Effusion as a Risk Marker

Oyama et al. identified minimal pleural or peritoneal fluid within 100 days before allogeneic HCT in 48 of 248 recipients. Pre-HCT effusion was associated with inferior overall survival after adjustment (hazard ratio, 1.85; 95% CI, 1.23–2.77) [7]. This finding supports pretransplant risk stratification; it does not estimate post-transplant pleural complication incidence.

### 3.3. Timing-Based Differential Diagnosis

Four intervals are used consistently: pre-HCT, days 0–30, days 31–100, and after day 100. Before HCT, malignancy, infection, hypoalbuminemia, cardiac or renal dysfunction, mediastinal lymphatic obstruction, central venous thrombosis, and tyrosine kinase inhibitor exposure are evaluated. During days 0–30, volume overload, bacterial or fungal infection, engraftment syndrome, capillary leak, sinusoidal obstruction syndrome, renal injury, cardiac dysfunction, transfusion-associated circulatory overload, and conditioning-related injury are prioritized [8,9].

During days 31–100, infection remains prominent, but idiopathic pneumonia syndrome, cytomegalovirus infection, transplant-associated thrombotic microangiopathy, drug toxicity, renal or cardiac dysfunction, and evolving GVHD enter the differential. After day 100, cGVHD serositis, organizing pneumonia-associated effusion, relapse or post-transplant lymphoproliferative disease, recurrent infection, drug-related effusion, and chylothorax should be considered. Timing narrows the differential but does not establish causality; unilateral, loculated, recurrent, rapidly enlarging, or unexplained effusions warrant a lower threshold for sampling. Table 1 aligns these intervals with evaluation and action.

## 4. Diagnostic Thoracentesis and Imaging

Direct thoracentesis data are limited. Adam et al. reported 50 thoracenteses in HCT recipients: 33 effusions (66%) were exudative, a specific diagnosis was established in 13 procedures (26%), 5 pneumothoraces occurred (10%), and no hemothorax was reported [10]. Bass and White reported 100 thoracenteses in patients with hematologic malignancy, including 21 HCT recipients; a specific diagnosis was established in 21%, with 7 pneumothoraces and 2 hemothoraces [11]. In a conference abstract, Nguyen et al. described 66 thoracenteses in 40 post-HCT patients without a reported procedure-related complication; this abstract-level evidence is supportive but not definitive [12].

Drainage should happen urgently when a large effusion causes respiratory compromise, tension physiology is suspected, or infected pleural fluid requires source control. Elective diagnostic thoracentesis should be performed for moderate or large unexplained effusion; unilateral, asymmetric, loculated, recurrent, or progressive fluid; suspected malignancy, relapse, or chylothorax; persistent sepsis; or uncertainty before increasing immunosuppression. Small bilateral effusions should be observed when a systemic cause is clinically compelling and the fluid improves promptly with treatment.

Bedside ultrasound before thoracentesis should be used to confirm a safe pocket, characterize septations, and distinguish fluid from pleural thickening or consolidation. Computed tomography should be used when loculation, pleural enhancement, mass, complex air–fluid anatomy, or mediastinal disease may change the approach. Color Doppler can help identify a variable intercostal artery, particularly posteriorly [13,14].

Standard testing should include pleural cell count and differential, protein and lactate dehydrogenase with paired serum values, glucose, and targeted microbiology. pH should be measured promptly in an anaerobic blood gas syringe when infection is suspected; bacterial cultures should be inoculated at the bedside when feasible. Cytology and flow cytometry are added for suspected hematologic malignancy or post-transplant lymphoproliferative disease; triglycerides, cholesterol, and chylomicrons for possible chylothorax; and fungal or mycobacterial studies are added only when epidemiology and immune deficits support them. Pleural N-terminal pro-B-type natriuretic peptide may support a cardiac cause but should not replace clinical and echocardiographic assessment [15].

Pleural fluid should be interpreted cautiously. Prior antimicrobials lower culture yield; diuresis can produce a biochemical pseudoexudate; hypoalbuminemia and systemic inflammation can blur Light’s criteria; and negative cytology does not exclude non-pleural relapse. Rare re-expansion pulmonary edema has been reported early after transplantation, but a single case does not justify a fixed lower drainage limit; use symptom- and pressure-aware drainage and stop for chest discomfort, marked cough, or instability [16].

**Figure 1:**
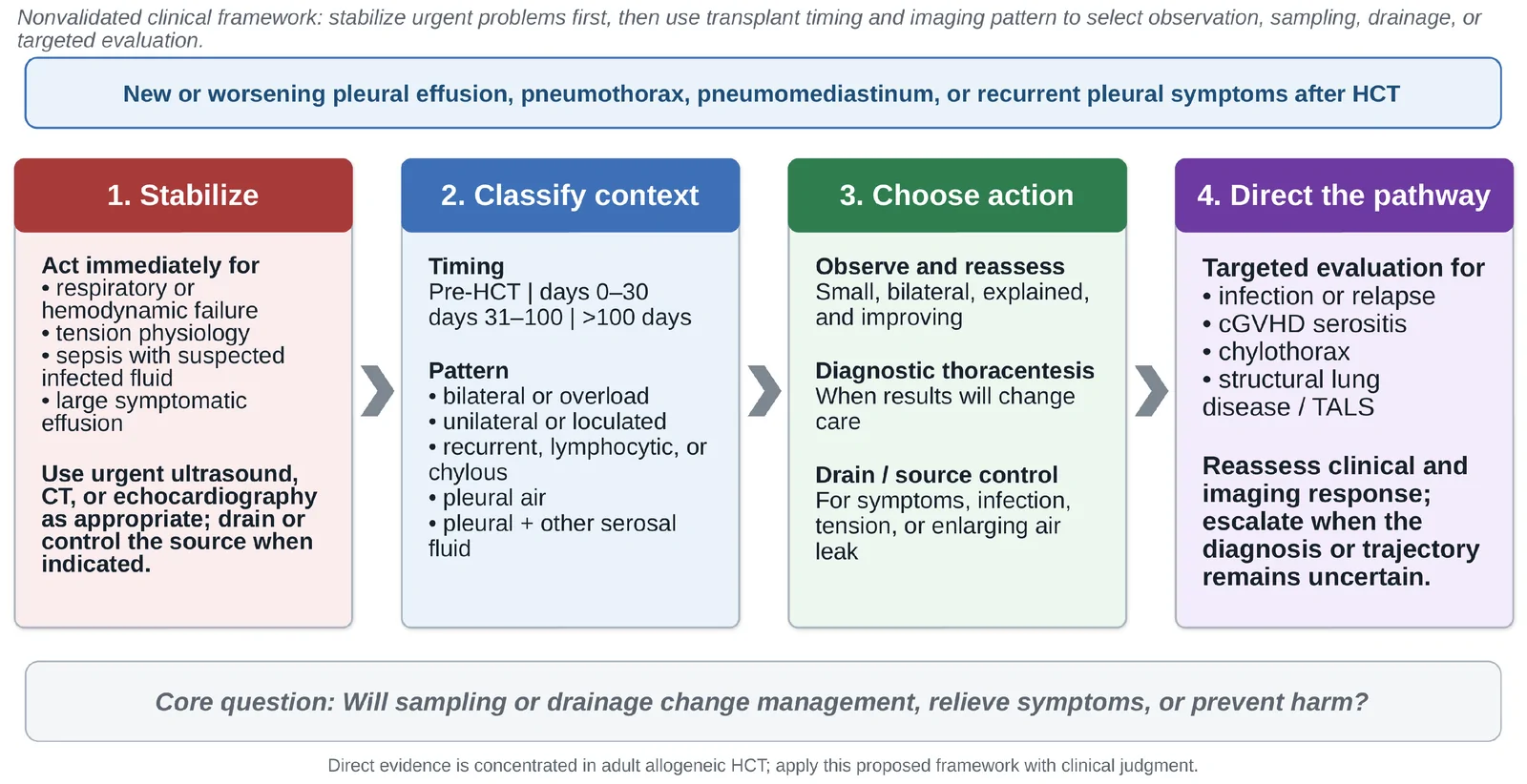
Proposed non-validated diagnostic framework for a new pleural effusion or thoracic air-leak abnormality after HCT. Urgent physiology should be stabilized first before transplant timing and radiographic pattern are used to select observation, diagnostic sampling, source control, or targeted evaluation. Direct evidence is concentrated in adult allogeneic HCT, so the framework requires clinical judgment.

Centers should standardize the indication, ultrasound findings, fluid panel, drainage stop criteria, and post-procedure monitoring. Figure 1 presents a proposed, non-validated diagnostic framework.

## 5. Chronic GVHD Serositis and Multi-Compartment Fluid

Under National Institutes of Health consensus terminology, serositis is an “other” or atypical manifestation and does not establish cGVHD by itself. Pleural cGVHD serositis should only be suspected in a compatible patient with diagnostic or distinctive cGVHD in another organ and after infection, relapse, cardiac or renal failure, hepatic disease, lymphatic obstruction, and drug toxicity have been excluded [17,18].

Historical and contemporary evidence remains observational. Seber et al. described unexplained multi-compartment effusions after allogeneic bone marrow transplantation; Leonard et al. reported cGVHD-associated serositis often after immunosuppression taper; and Doering et al. found serositis or polyserositis in 2.2% of 623 allogeneic HCT recipients [19,20,21]. In the largest modern cohort, Te et al. identified 50 adults with symptomatic serositis attributed to cGVHD; 38 had pleural effusions. Most received intensified immunosuppression, but pleural fluid data were insufficient to define a diagnostic profile or agent-specific response [22].

Because pleural and pericardial involvement can coexist, one should obtain echocardiography when symptoms, examination, or imaging suggest multi-compartment serosal inflammation; detailed pericardial epidemiology is outside the scope of this review [22,23,24]. Drug toxicity should be identified by exposure timing, recurrence with rechallenge when known, and improvement after dose reduction or withdrawal; cGVHD attribution requires the broader NIH clinical context.

Treat symptomatic fluid with diagnostic or therapeutic drainage when indicated and coordinate systemic therapy with the transplant team. Case reports describe corticosteroid-responsive and steroid-refractory serositis, but they do not establish a sequence [25,26]. For steroid-refractory cGVHD overall, ruxolitinib improved the primary week-24 overall response (49.7% vs. 25.6%) and failure-free survival compared with best available therapy in REACH3; that trial was not serositis-specific [27,28,29]. Later-line therapy should be selected according to overall cGVHD burden, cytopenias, infection risk, prior exposure, and patient goals. Table 2 summarizes management strategies without implying serositis-specific randomized efficacy.

## 6. Thoracic Air-Leak Syndrome

TALS is an umbrella term for post-HCT air outside the normal tracheobronchial lumen. Pneumothorax contains pleural air; pneumomediastinum contains mediastinal air; pneumopericardium contains pericardial air; and subcutaneous or pulmonary interstitial emphysema are extraplural manifestations. These entities may coexist but should not be conflated with pleural effusion [30,31,32,33].

Outcomes are poor in reported cohorts. Moon et al. identified 18 cases among 2177 allogeneic HCT recipients (0.83%). Sakai et al. identified 18 cases among 1515 adults and reported 3-year survival of 14.9% compared with 61.5% in recipients without air-leak syndrome. In the pediatric series by Zhao et al., 10 of 28 patients died from respiratory failure or infection [30,31,33]. These estimates come from retrospective cohorts with heterogeneous definitions.

Risk factors and mechanisms differ from treatment decisions. BOS can cause severe air trapping and alveolar rupture; pleuroparenchymal fibroelastosis (PPFE) and restrictive pulmonary cGVHD can predispose to recurrent pneumothorax. Infection, cough, positive-pressure ventilation, and fragile corticosteroid-exposed tissue may contribute, but causal pathways are not fully established [32,34].

Selected small, stable air leaks should be observed with close imaging and oxygen as clinically appropriate. A pleural drain should be inserted for tension physiology, enlarging or symptomatic pneumothorax, respiratory compromise, or failure of conservative management. Early interventional pulmonary or thoracic surgical review should be requested for persistent air leak over several days, recurrent pneumothorax, failure of lung re-expansion, suspected bronchopleural fistula, or inability to reduce ventilatory support. Options include pleurodesis, autologous blood patch, bronchoscopic valves, or surgery; published HCT experience is limited, and interventions may control the leak without altering progressive pulmonary cGVHD [35,36].

Transbronchial biopsy and transbronchial lung cryobiopsy are not pleural diagnostic tests. Their relevance is iatrogenic pneumothorax risk in patients with limited reserve; pediatric American Thoracic Society guidance discourages transbronchial biopsy for BOS diagnosis [37]. Parenchymal biopsy should be reserved for a specific management change query after multidisciplinary review.

## 7. Chylothorax, Drug-Related Effusion, and Cellular Therapy

Chylothorax after HCT lacks reliable incidence data. It is diagnosed by demonstrating chylomicrons or, in the appropriate context, pleural triglycerides above 110 mg/dL. A value below 50 mg/dL makes chylothorax unlikely, although fasting or malnutrition can lower triglycerides; chylomicrons should be obtained when values are intermediate or suspicion remains high. Pseudochylothorax instead reflects chronic cholesterol-rich fluid, typically with pleural cholesterol above 200 mg/dL or cholesterol crystals [38,39].

Management should begin with the underlying cause and the patient’s respiratory and nutritional status, and drainage should be used only as needed for symptoms or respiratory compromise; a low-fat diet enriched with medium-chain triglycerides and dietitian support should be implemented; and temporary parenteral nutrition or octreotide should be considered in selected persistent cases. During ongoing high-volume loss, drainage volume, weight, albumin and total protein, electrolytes, fat-soluble vitamins and other micronutrients, lymphocyte count, and infection risk should be monitored.

Escalation to lymphangiography and possible thoracic duct embolization (TDE) should occur when high-output drainage persists, conservative therapy fails, recurrent fluid causes respiratory compromise, or nutritional and immune losses accumulate. No HCT-specific output or duration threshold has been validated, so interventional radiology should be involved early when definitive treatment may be required. Contemporary guidance and lymphoma series support lymphatic imaging and TDE in refractory non-traumatic chylothorax [38,39,40].

Dasatinib is an established cause of exudative pleural effusion and rare chylothorax. Temporal exposure, exclusion of competing causes, and improvement after interruption or dose reduction should be used (rather than speculative mechanisms) to support attribution [49,50,51,52].

CAR-T therapy is not HCT. Effusions around CAR-T may reflect baseline lymphoma, infection, heart failure, capillary leak, or cytokine release syndrome (CRS) [53,54,55]. CRS can be graded using American Society for Transplantation and Cellular Therapy (ASTCT) criteria based on fever, hypotension, and hypoxia; pleural effusion itself is not a CRS grading criterion [56]. We include this adjacent evidence only to inform differential diagnosis in shared transplant and cellular therapy programs.

## 8. Pleural Infection and Recurrent Effusion

Pleural infection after HCT is undercharacterized. In two selected thoracentesis cohorts, empyema was found in 1 of 50 HCT procedures and 1 of 100 hematologic malignancy procedures; these proportions describe sampled effusions and are not incidence estimates for all HCT recipients [10,11]. Bacterial cultures should be obtained routinely when infection is suspected and fungal or mycobacterial studies should be added according to immune deficit, imaging, prophylaxis, and exposure history.

Source control should be pursued for frank pus, positive Gram stain or culture, pleural pH at or below 7.20 in a compatible clinical setting, loculation or enlarging collection, or persistent sepsis despite antimicrobials. Image-guided small-bore drainage should be used when feasible [41]. Tissue plasminogen activator plus deoxyribonuclease for residual loculated infection may be considered after adequate tube placement. In a large general-population cohort, pleural bleeding occurred in approximately 4%; therapeutic anticoagulation and platelet counts below 100 × 10^9^/L were associated with greater risk [42,43,44]. HCT-specific efficacy and platelet thresholds do not exist; IET should be avoided during active bleeding, and therapeutic anticoagulation should be interrupted when clinically feasible.

For recurrent symptomatic effusion, a choice between serial thoracentesis, an indwelling pleural catheter (IPC), pleurodesis, and conservative management must be made. Some studies use the term tunneled pleural catheter for the same device category; this review uses IPC consistently. In 172 hematologic malignancy patients with IPCs, empyema occurred in 2.9%, major bleeding in 1.7%, and 30-day procedure-associated mortality in 0.6%; these are not HCT-specific estimates [45,46]. Multicenter data suggest antineoplastic therapy alone does not markedly increase IPC infection risk, but early post-HCT cytopenia, bacteremia, mucositis, skin integrity, lung expandability, home support, and prognosis require individual assessment [47,48].

## 9. Procedure-Specific Safety

A procedure-specific hemostatic approach should be used rather than one threshold for all pleural interventions. For ultrasound-guided thoracentesis, observational cohorts and a systematic review show very low major bleeding despite selected uncorrected coagulation abnormalities [57,58,59]. CHEST conditionally advises against routine prophylactic platelet or fresh frozen plasma transfusion before bedside thoracentesis or paracentesis in critically ill adults; certainty is very low [60]. SIR classifies thoracentesis as a low-bleeding-risk image-guided procedure and uses a platelet count of at least 20 × 10^9^/L as a reference point [61]. Neither statement is an HCT-specific safety guarantee. Table 3 summarizes the core evidence base, study design, and applicability.

For small-bore chest tubes and IPC placement, one must account for greater tissue disruption, the ability to compress or rescue bleeding, infection risk, and procedural urgency. No evidence-based HCT-specific platelet threshold has been established. For non-urgent tunneled catheter placement, some centers use a platelet count of at least 50 × 10^9^/L as a pragmatic institutional target when feasible; this is extrapolated practice rather than a validated safety threshold and should not delay lifesaving drainage. Barkan et al. reported nine tube thoracostomies in four allogeneic HCT recipients without bleeding or pleural infection. Madenci et al. reported eight pediatric HCT recipients with pleural drains and no drain-related bleeding or infection [62,63].

**Figure 2:**
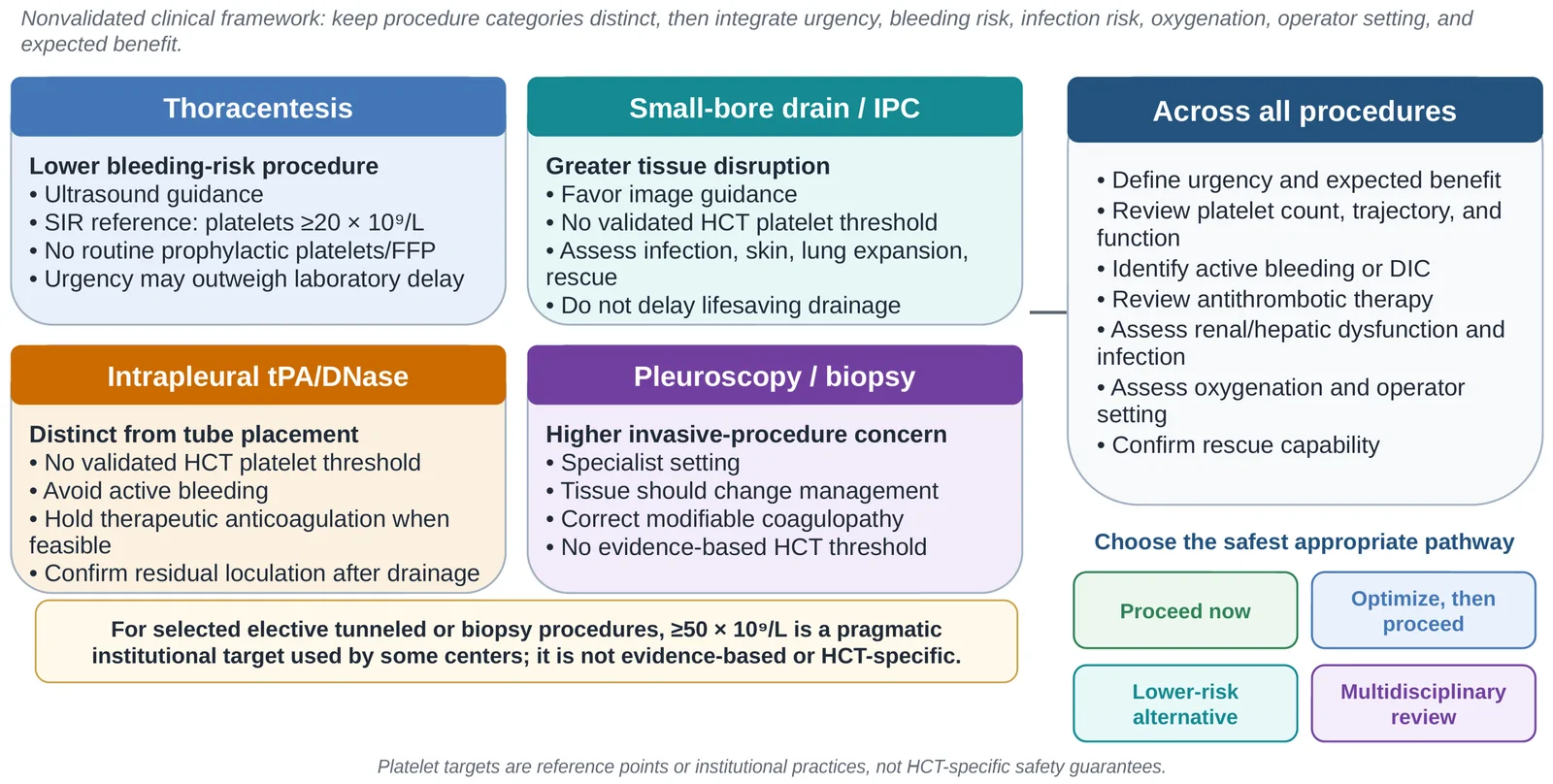
Proposed, non-validated procedure-specific safety framework for pleural interventions after HCT. Intrapleural tPA/DNase is shown as a distinct pathway from pleuroscopy or biopsy because the procedures have different evidence bases and bleeding risk considerations. The platelet count of at least 50 × 10^9^/L shown for selected elective tunneled or biopsy procedures is a pragmatic institutional reference used by some centers; it is not an evidence-based or HCT-specific threshold. Urgency, platelet trajectory and function, active bleeding, antithrombotic therapy, organ dysfunction, infection, oxygenation, operator setting, and rescue capability should determine whether to proceed, optimize, choose an alternative, or obtain multidisciplinary review.

For pleuroscopy or pleural biopsy, a specialist setting should be used. Some centers use a platelet count of at least 50 × 10^9^/L as a pragmatic institutional target for these elective higher-risk procedures, but the value is not evidence-based or HCT-specific. Correct active coagulopathy when possible and proceed only when tissue will change management. IET should be considered separately from biopsy-based procedures: the infection-specific precautions described above should be used, with no validated HCT platelet threshold. Figure 2 and Table 4 provide proposed, non-validated procedure-specific frameworks.

## 10. Practical Recommendations

- Stabilize respiratory or hemodynamic compromise before pursuing etiologic classification.
- Use transplant phase and radiographic pattern to prioritize the differential, but do not infer cause from timing alone.
- Use ultrasound for every bedside thoracentesis and image guidance for small, loculated, posterior, or otherwise high-risk collections.
- Perform thoracentesis when sampling will change management, symptoms require relief, or infection, malignancy, chylothorax, or serositis remains uncertain.
- Diagnose cGVHD serositis only after applying NIH terminology and excluding infection, relapse, organ failure, lymphatic obstruction, and drug toxicity.
- Escalate TALS care early for tension physiology, recurrent pneumothorax, failure of lung re-expansion, persistent air leak, or ventilatory dependence.
- Treat chylothorax with cause-directed therapy, nutritional support, loss monitoring, and timely lymphatic intervention when conservative management fails.
- Apply procedure-specific platelet reference points and bleeding precautions; do not use one threshold for thoracentesis, drains, IPCs, IET, and pleural biopsy.
- Document the indication, imaging guidance, hemostatic context, procedural events, and 30-day outcomes to strengthen the HCT-specific evidence base.

## 11. Limitations and Research Priorities

This review is narrative, not systematic. It lacks preregistration, duplicate screening, formal risk-of-bias assessment, and quantitative synthesis; source selection bias is therefore possible. Direct evidence is dominated by retrospective adult allogeneic HCT cohorts. Pediatric, autologous HCT, haploidentical HCT, and contemporary cellular therapy data are sparse. Definitions of clinically significant effusion, serositis, TALS, and procedural complications vary, and most hemostatic and IPC recommendations are extrapolated from non-HCT populations. The proposed figures synthesize clinical reasoning but have not been prospectively validated.

Prospective registries should use explicit denominators and standardized definitions and record transplant platform and phase, radiographic size and distribution, pleural fluid phenotype, platelet count and function, anticoagulant exposure, ultrasound or image guidance, procedure type and urgency, transfusion strategy, microbiology, 7- and 30-day bleeding, pneumothorax and infection events, need for repeat intervention, and 90-day survival. Serositis studies should also define NIH cGVHD context and organ-specific response. These targets would permit procedure-specific risk models rather than additional undifferentiated case series.

## 12. Conclusions

Pleural and thoracic air-leak complications after HCT require an evidence-calibrated, procedure-specific approach. First, urgent physiology should be stabilised before transplant timing and imaging pattern are utilized to decide whether observation, thoracentesis, source control, or targeted evaluation is most likely to change care. cGVHD serositis should be treated as a diagnosis of exclusion, and the nutritional and immune consequences of chylothorax should be monitored and treated. TALS management should be escalated when the air leak persists or compromises ventilation. For pleural procedures, ultrasound or image guidance can be combined with individualized hemostatic assessment rather than applying one platelet threshold to every intervention. Until prospective data are available, clinicians should document standardized procedural and 30-day outcomes so routine care can build an HCT-specific evidence base.

## Figures and Tables

**Table 1:** Timing-based differential diagnosis of pleural effusion after HCT.

Interval	Priority	Clinical Pattern	Recommended Evaluation	Observation vs. Sampling/Drainage
Pre-HCT	Malignancy; infection; hypoalbuminemia; cardiac/renal dysfunction; venous or lymphatic obstruction; TKI toxicity	Baseline fluid; active disease; lymphadenopathy; fever; low albumin; drug exposure	Review baseline CT; assess volume status and infection; echocardiography when indicated	Observe a small explained effusion; sample a moderate/large, unilateral, symptomatic, progressive, or unexplained effusion
Days 0–30	Volume overload; bacterial/fungal infection; engraftment syndrome; capillary leak; sinusoidal obstruction syndrome; renal/cardiac injury; transfusion overload	Weight gain; neutropenia; fever; mucositis; bilirubin or creatinine rise; peri-engraftment hypoxemia	Bedside ultrasound; cultures; CT for suspected infection/loculation; trial of diuresis only when overload is compelling	Observe small bilateral fluid that promptly improves; drain for respiratory compromise, infected fluid, or tension; sample unilateral/loculated/non-resolving fluid
Days 31–100	Infection; idiopathic pneumonia syndrome; CMV; TMA; drug toxicity; renal/cardiac dysfunction; evolving GVHD	Persistent hypoxemia; viral reactivation; hemolysis/TMA markers; organ dysfunction; new asymmetry or loculation	CT chest; infectious testing; echocardiography; TMA evaluation; thoracentesis if diagnosis will change management	Sample unexplained, unilateral, progressive, recurrent, or febrile effusion and before increasing immunosuppression when infection/relapse remains plausible
>100 days	cGVHD serositis; organizing pneumonia-associated effusion; relapse/PTLD; recurrent infection; drug toxicity; chylothorax	Established cGVHD; immunosuppression taper; other serosal fluid; recurrent/lymphocytic/chylous effusion; pleural air	Thoracentesis with cytology/flow or triglycerides when indicated; echocardiography; CT; pulmonary function testing after stabilization	Lower threshold to sample recurrent, unilateral, chylous, loculated, or multi-compartment fluid; drain symptomatic fluid or pleural infection

Abbreviations: cGVHD, chronic graft-versus-host disease; CMV, cytomegalovirus; CT, computed tomography; GVHD, graft-versus-host disease; HCT, hematopoietic cell transplantation; PTLD, post-transplant lymphoproliferative disease; TKI, tyrosine kinase inhibitor; TMA, thrombotic microangiopathy.

**Table 2:** Management strategies for pleural and thoracic air-leak complications after HCT.

Entity	Diagnostic Trigger	Initial Management	Escalation Trigger/Option	Evidence Boundary
cGVHD serositis	Late or recurrent effusion with established cGVHD; often after immunosuppression taper; competing causes excluded	Diagnostic/therapeutic drainage when indicated; echocardiography if multi-compartment involvement suspected; coordinate systemic cGVHD therapy	Escalate systemic cGVHD therapy for steroid-refractory disease with the transplant team	Direct HCT series exist, but there are no serositis-specific randomized therapy data [17,18,19,20,21,22,23,24,25,26,27,28,29]
Thoracic air-leak syndrome	Pneumothorax, pneumomediastinum, pneumopericardium, subcutaneous or interstitial emphysema after HCT	Stabilize; oxygen/observation for selected stable cases; pleural drain for tension, enlarging or symptomatic pneumothorax	Early IP/surgery review for recurrent pneumothorax, persistent leak, failed re-expansion, fistula, or ventilatory dependence; consider pleurodesis, blood patch, valves, or surgery	Direct retrospective adult/pediatric HCT cohorts; heterogeneous definitions; no validated management algorithm [30,31,32,33,34,35,36,37]
Chylothorax	Chylomicrons or triglycerides > 110 mg/dL; consider despite lower triglycerides in fasting/malnutrition	Treat cause; symptom-directed drainage; low-fat/MCT diet; dietitian; monitor fluid, protein, electrolyte, micronutrient, and lymphocyte losses	Lymphangiography ± TDE for persistent high output, failed conservative therapy, recurrent respiratory compromise, or cumulative nutritional/immune loss	No HCT incidence or validated escalation threshold; guidance extrapolated from non-traumatic and lymphoma-associated chylothorax [38,39,40]
Pleural infection	Pus/positive culture; pH ≤ 7.20 in compatible setting; loculation/enlargement; persistent sepsis	Antimicrobials plus image-guided small-bore source control	Consider tPA/DNase for residual loculation; surgery when drainage/IET fails or anatomy demands it	No HCT-specific IET trial; bleeding data extrapolated from general cohorts [41,42,43,44]
Recurrent symptomatic effusion	Repeated symptomatic recurrence after cause-directed therapy; non-expandable lung; malignant/chylous fluid; persistent serositis	Serial thoracentesis, IPC, pleurodesis, or conservative care according to prognosis and lung expandability	IPC when repeated outpatient drainage is likely; pleurodesis when lung expands and durable symphysis is desired	IPC safety data derive mainly from hematologic malignancy and immunosuppressed cohorts, not dedicated HCT cohorts [45,46,47,48]

Abbreviations: cGVHD, chronic graft-versus-host disease; HCT, hematopoietic cell transplantation; IET, intrapleural enzyme therapy; IP, interventional pulmonology; IPC, indwelling pleural catheter; MCT, medium-chain triglyceride; TDE, thoracic duct embolization; tPA/DNase, tissue plasminogen activator/deoxyribonuclease.

**Table 3:** Core evidence base, study design, and applicability.

Study/Domain	Population	Main Results	Study Design/Source Type	Directness	Key Limitation
Modi et al. [4]	618 adults after allogeneic HCT; 71 clinically significant effusions	1-year cumulative incidence 9.9% (95% CI, 7.7–12.5%); median onset day 40; bimodal timing	Single-center retrospective cohort	Direct adult allogeneic HCT	Clinically recognized events; may miss small radiographic effusions
Masuda et al. [5]	178 first allogeneic HCT recipients; 106 new pleural effusions	Day-100 cumulative incidence 41.0%; 2-year survival 18.8% with three involved cavities	Single-center retrospective CT review	Direct adult allogeneic HCT	Imaging-detected fluid; not directly comparable with clinically significant cohorts
Oyama et al. [7]	248 first allogeneic HCT recipients; 48 with minimal pre-HCT fluid	Adjusted hazard ratio for overall survival 1.85 (95% CI, 1.23–2.77)	Single-center retrospective cohort	Direct pre-HCT risk-marker evidence	Not a post-HCT incidence study
Adam et al. [10]	50 thoracenteses in HCT recipients	Specific diagnosis 13/50 (26%); pneumothorax 5/50; hemothorax 0/50	Retrospective HCT procedure cohort	Direct HCT	Small, older cohort; limited modern microbiology and ultrasound
Bass and White [11]	100 thoracenteses in hematologic malignancy; 21 HCT recipients	Specific diagnosis 21/100; pneumothorax 7/100; hemothorax 2/100	Retrospective procedure cohort	Partly direct; mostly adjacent	Mixed malignancy population; events not stratified fully by HCT
Faiz et al. [9]	111 acute leukemia/MDS patients; 270 pleural procedures	43% of procedures had platelets < 20 × 10^9^/L; complications 1.9% after clinical optimization	Single-center retrospective cohort	Extrapolated hematologic malignancy	Not HCT; transfusion/optimization limits inference about uncorrected counts
Te et al. [22]	50 adults with cGVHD serositis; 38 pleural effusions	Most received intensified immunosuppression; pleural fluid phenotype and agent-specific response not defined	Single-center retrospective cGVHD cohort	Direct HCT	Diagnosis by clinical attribution; no prospective response criteria
Moon/Sakai/Zhao [30,31,33]	Adult and pediatric allogeneic HCT cohorts	Moon: 18/2177; Sakai: 3-year survival 14.9%; Zhao: 10/28 deaths	Retrospective cohorts	Direct HCT	Rare events; heterogeneous definitions and treatment
Faiz IPC cohort [46]	172 hematologic malignancy patients with IPCs	Empyema 2.9%; major bleeding 1.7%; 30-day procedure mortality 0.6%	Single-center retrospective cohort	Extrapolated hematologic malignancy	Not dedicated HCT; referral and selection bias
CHEST/SIR/ERS/ESTS guidance [41,60,61]	General critical care, interventional radiology, and pleural populations	Procedure-specific transfusion, platelet, source control, and IET guidance	Society guidelines/consensus	Extrapolated procedural guidance	Not developed or validated specifically for HCT

Abbreviations: cGVHD, chronic graft-versus-host disease; CI, confidence interval; CT, computed tomography; HCT, hematopoietic cell transplantation; IPC, indwelling pleural catheter; IET, intrapleural enzyme therapy; MDS, myelodysplastic syndrome; SIR, Society of Interventional Radiology.

**Table 4:** Procedure-specific safety considerations after HCT.

Procedure	Observed Data/Events	Reference Point or Risk Category	HCT-Specific Actions and Cautions
Thoracentesis	HCT cohort: 5 pneumothoraces and 0 hemothoraces in 50 procedures [10]. Hematologic malignancy cohort: 7 pneumothoraces and 2 hemothoraces in 100 procedures [11]. Major bleeding was rare in general coagulopathy studies [57,58,59].	Low bleeding risk with ultrasound. SIR reference: platelets ≥ 20 × 10^9^/L [61]. CHEST advises against routine prophylactic platelets/FFP (conditional; very low certainty) [60].	Use ultrasound; do not delay urgent therapeutic drainage solely for an isolated count; account for platelet trend/function, active bleeding/DIC, anticoagulants, oxygenation, and rescue capability.
Image-guided small-bore chest tube	Barkan: 9 drains in 4 allogeneic HCT recipients, with 0 bleeding and 0 pleural/local infection [62]. Madenci: 8 pediatric HCT recipients with pleural drains, with 0 drain-related bleeding or infection [63].	Greater tissue disruption than thoracentesis. No validated HCT platelet threshold; correct modifiable hemostatic abnormalities when feasible without delaying urgent source control.	Use image guidance when possible; prioritize source control or tension relief; monitor for hemothorax, infection, tube obstruction, and persistent air leak.
Indwelling pleural catheter	Faiz: 172 hematologic malignancy patients; empyema 2.9%, major bleeding 1.7%, 30-day procedure mortality 0.6% [46].	Tunneled procedure. Some centers use platelets ≥ 50 × 10^9^/L as a pragmatic institutional target for elective placement; this is extrapolated practice, not an evidence-based or HCT-specific threshold.	Exclude uncontrolled pleural infection; assess skin integrity, bacteremia, lung expandability, home drainage capacity, prognosis, and goals.
Intrapleural tPA/DNase	General cohort pleural bleeding approximately 4%; therapeutic anticoagulation and platelets < 100 × 10^9^/L associated with higher risk [43].	No validated HCT platelet threshold. Treat as a distinct bleeding-risk decision, separate from tube placement and from pleuroscopy or biopsy.	Avoid active bleeding; hold therapeutic anticoagulation when feasible; confirm tube position and residual loculation; involve surgery early if source control remains inadequate.
Pleuroscopy or pleural biopsy	No dedicated HCT safety cohort.	Higher invasive-procedure concern. Some centers use platelets ≥ 50 × 10^9^/L as a pragmatic institutional target; this is not an evidence-based or HCT-specific threshold.	Use a specialist setting; correct modifiable coagulopathy; proceed only when tissue is expected to change management and less invasive tests are insufficient.

Note: A platelet count of at least 50 × 10^9^/L is presented only as a pragmatic institutional target used by some centers for selected elective tunneled or biopsy procedures; it is not an evidence-based or HCT-specific threshold. Abbreviations: DIC, disseminated intravascular coagulation; FFP, fresh frozen plasma; HCT, hematopoietic cell transplantation; SIR, Society of Interventional Radiology; tPA/DNase, tissue plasminogen activator/deoxyribonuclease.

## Data Availability

No new data were created or analyzed in this study. Data sharing is not applicable to this article.

## Abbreviations

ASTCT, American Society for Transplantation and Cellular Therapy; BOS, bronchiolitis obliterans syndrome; CAR-T, chimeric antigen receptor T-cell therapy; cGVHD, chronic graft-versus-host disease; CI, confidence interval; CMV, cytomegalovirus; CRS, cytokine release syndrome; CT, computed tomography; DIC, disseminated intravascular coagulation; FFP, fresh frozen plasma; GVHD, graft-versus-host disease; HCT, hematopoietic cell transplantation; IET, intrapleural enzyme therapy; IPC, indwelling pleural catheter; LDH, lactate dehydrogenase; MCT, medium-chain triglyceride; PPFE, pleuroparenchymal fibroelastosis; SIR, Society of Interventional Radiology; TALS, thoracic air-leak syndrome; TDE, thoracic duct embolization; TKI, tyrosine kinase inhibitor; TMA, thrombotic microangiopathy.

## References

[1] Shiari A., Nassar M., Soubani A.O. (2021). Major pulmonary complications following hematopoietic stem cell transplantation: What the pulmonologist needs to know. Respir. Med..

[2] Bertini C., Soto F., Brown R., Bashoura L., Cheng G.-S., Hensley M.K., Hsu J., Lai Y.K., Sharifi H., Sturek J.M. (2025). Noninfectious pulmonary complications after hematopoietic cell transplantation: A comprehensive review. Respir. Med..

[3] Patel S.S., Ahn K.W., Khanal M., Bupp C., Allbee-Johnson M., Majhail N.S., Hamilton B.K., Rotz S.J., Hashem H., Beitinjaneh A. (2022). Noninfectious pulmonary toxicity after allogeneic hematopoietic cell transplantation. Transplant. Cell. Ther..

[4] Modi D., Jang H., Kim S., Deol A., Ayash L., Bhutani D., Lum L.G., Ratanatharathorn V., Manasa R., Mellert K. (2016). Incidence, etiology, and outcome of pleural effusions in allogeneic hematopoietic stem cell transplantation. Am. J. Hematol..

[5] Masuda Y., Honda A., Oyama T., Masamoto Y., Kurokawa M. (2025). Prognostic impact of effusion in multiple body cavities after allogeneic hematopoietic stem cell transplantation. Int. J. Hematol..

[6] Baethge C., Goldbeck-Wood S., Mertens S. (2019). SANRA-a scale for the quality assessment of narrative review articles. Res. Integr. Peer Rev..

[7] Oyama T., Honda A., Masuda Y., Morita K., Maki H., Masamoto Y., Kurokawa M. (2025). Pretransplant minimal pleural and peritoneal effusion is a potential poor prognostic indicator in allogeneic hematopoietic stem cell transplantation. Clin. Transplant..

[8] Alexandrakis M.G., Passam F.H., Kyriakou D.S., Bouros D. (2004). Pleural effusions in hematologic malignancies. Chest.

[9] Faiz S.A., Bashoura L., Lei X., Sampat K.R., Brown T.C., Eapen G.A., Morice R.C., Ferrajoli A., Jimenez C.A. (2013). Pleural effusions in patients with acute leukemia and myelodysplastic syndrome. Leuk. Lymphoma..

[10] Adam A.K., Zamlut M., Soubani A.O. (2007). The yield and safety of thoracentesis in hematopoietic stem cell transplantation recipients. Lung.

[11] Bass J., White D.A. (2005). Thoracentesis in patients with hematologic malignancy: Yield and safety. Chest.

[12] Nguyen A., Bashoura L., Jimenez C.A., Morice R.C., Ost D.E., Eapen G.A., Faiz S.A. (2010). Characteristics of pleural effusions in patients after hematopoietic stem cell transplantation. Chest.

[13] Sundaralingam A., Grabczak E.M., Burra P., Costa M.I., George V., Harriss E., Jankowska E.A., Janssen J.P., Karpathiou G., Laursen C.B. (2024). ERS statement on benign pleural effusions in adults. Eur. Respir. J..

[14] Salamonsen M., Ellis S., Paul E., Steinke K., Fielding D. (2012). Thoracic ultrasound demonstrates variable location of the intercostal artery. Respiration.

[15] Yeh J.-H., Huang C.-T., Liu C.-H., Ruan S.-Y., Tsai Y.-J., Chien Y.-C., Yang C.-Y., Hsu C.-L., Kuo L.-C., Lee P.-L. (2014). Cautious application of pleural N-terminal pro-B-type natriuretic peptide in diagnosis of congestive heart failure pleural effusions among critically ill patients. PLoS ONE.

[16] Garantziotis S., Bhalla K.S., Long G.D., Vredenburgh J.J., Folz R.J. (2002). Fatal re-expansion pulmonary edema associated with increased lung IL-8 levels following high-dose chemotherapy and autologous stem cell transplant. Respiration.

[17] Jagasia M.H., Greinix H.T., Arora M., Williams K.M., Wolff D., Cowen E.W., Palmer J., Weisdorf D., Treister N.S., Cheng G.-S. (2015). National Institutes of Health consensus development project on criteria for clinical trials in chronic graft-versus-host disease: I. The 2014 Diagnosis and Staging Working Group report. Biol. Blood Marrow Transplant..

[18] Cuvelier G.D.E., Schoettler M., Buxbaum N.P., Pinal-Fernandez I., Schmalzing M., Distler J.H., Penack O., Santomasso B.D., Zeiser R., Angstwurm K. (2022). Toward a better understanding of the atypical features of chronic graft-versus-host disease: A report from the 2020 National Institutes of Health Consensus Project Task Force. Transplant. Cell. Ther..

[19] Seber A., Khan S.P., Kersey J.H. (1996). Unexplained effusions: Association with allogeneic bone marrow transplantation and acute or chronic graft-versus-host disease. Bone Marrow Transpl..

[20] Leonard J.T., Newell L.F., Meyers G., Hayes-Lattin B., Gajewski J., Heitner S., Nonas S., Allen B., Stentz A., Frires R. (2015). Chronic GvHD-associated serositis and pericarditis. Bone Marrow Transpl..

[21] Doering J., Perl M., Weber D., Banas B., Schulz C., Hamer O.W., Angstwurm K., Holler E., Herr W., Edinger M. (2023). Incidence and outcome of atypical manifestations of chronic graft-versus-host disease: Results from a retrospective single-center analysis. Transplant. Cell. Ther..

[22] Te C., Elahi T., Samples K., Onstad L., Lee S.J. (2025). Serositis associated with chronic graft-versus-host disease. Transplant. Cell. Ther..

[23] Kubo H., Imataki O., Fukumoto T., Oku M., Ishida T., Kubo Y.H., Kida J.-I., Uemura M., Fujita H., Ohno H. (2021). Risk factors for and the prognostic impact of pericardial effusion after allogeneic hematopoietic stem cell transplantation. Transplant. Cell. Ther..

[24] Tinianow A., Gay J.C., Bearl D.W., Connelly J.A., Godown J., Kitko C.L. (2020). Pericardial effusion following hematopoietic stem cell transplantation in children: Incidence, risk factors, and outcomes. Pediatr. Transplant..

[25] Masuda Y., Yamazaki S., Honda A., Masamoto Y., Kurokawa M. (2024). Isolated massive pleural effusion as a manifestation of chronic graft versus host disease successfully treated with corticosteroid. Ann. Hematol..

[26] Xiong Z., Wang Z., Chen X. (2025). Case report: Diagnosis and successful treatment of a rare case of steroid-refractory chronic graft-vs-host disease-related serositis. Front. Immunol..

[27] Ye Y., Savani B., Malard F., Ruggeri A., Aboudale I., Peric Z., Huang H., Mohty M. (2026). Chronic graft-versus-host disease. Nat. Rev. Dis. Primers.

[28] Loren A.W., Mielcarek M., Bolaños-Meade J., Brammer J., Cowden M., Di Stasi A., El-Jawahri A., Elmariah H., Gundabolu K., Gutman J. (2025). Hematopoietic Cell Transplantation, Version 3.2025, NCCN Clinical Practice Guidelines in Oncology. J. Natl. Compr. Canc Netw..

[29] Zeiser R., Polverelli N., Ram R., Hashmi S.K., Chakraverty R., Middeke J.M., Musso M., Giebel S., Uzay A., Langmuir P. (2021). Ruxolitinib for glucocorticoid-refractory chronic graft-versus-host disease. N. Engl. J. Med..

[30] Moon M.H., Sa Y.J., Cho K.D., Jo K.H., Lee S.H., Sim S.B. (2010). Thoracic air-leak syndromes in hematopoietic stem cell transplant recipients with graft-versus-host disease: A possible sign for poor response to treatment and poor prognosis. J. Korean Med. Sci..

[31] Sakai R., Kanamori H., Nakaseko C., Yoshiba F., Fujimaki K., Sakura T., Fujisawa S., Kawai N., Onoda M., Matsushima T. (2011). Air-leak syndrome following allo-SCT in adult patients: Report from the Kanto Study Group for Cell Therapy in Japan. Bone Marrow Transpl..

[32] Liu Y.C., Chou Y.H., Ko P.S., Wang H.-Y., Fan N.-W., Liu C.-J., Hsiao L.-T., Chien S.-H., Chiou T.-J., Liu J.-H. (2019). Risk factors and clinical features for post-transplant thoracic air-leak syndrome in adult patients receiving allogeneic haematopoietic stem cell transplantation. Sci. Rep..

[33] Zhao W., Wang R., Chen M. (2024). Clinical analysis of air-leak syndrome following allogeneic hematopoietic stem cell transplantation in pediatric patients. Pediatr. Blood Cancer..

[34] Bos S., Beeckmans H., Vanstapel A., Sacreas A., Geudens V., Willems L., Schreurs I., Vanaudenaerde B.M., Schoemans H., Vos R. (2022). Pulmonary graft-versus-host disease and chronic lung allograft dysfunction: Two sides of the same coin?. Lancet Respir. Med..

[35] Persano G., Crocoli A., Martucci C., Pardi V., Di Paolo P.L., Petreschi F., Cafiero G., Inserra A. (2023). Recurrent thoracic air leak syndrome in patients affected by pulmonary graft-versus-host disease: Surgical strategies and outcome. Pediatr. Pulmonol..

[36] Smesseim I., Morin-Thibault L.-V., Herth F.J.F., Tonkin J., Shah P.L., Slebos D.-J., Koster D.T., Dickhoff C., Daniels J.M.A., Annema J. (2024). Endobronchial valves in treatment of persistent air leak: European case-series study and best practice recommendations—From an expert panel. Respiration.

[37] Shanthikumar S., Gower W.A., Srinivasan S., Rayment J.H., Robinson P.D., Bracken J., Stone A., Das S., Barochia A., Charbek E. (2024). Detection of bronchiolitis obliterans syndrome after pediatric hematopoietic stem cell transplantation: An official American Thoracic Society clinical practice guideline. Am. J. Respir. Crit. Care Med..

[38] Monroe E.J., Kim C.Y., Ahmed O., Cline B., Elliott I.A., Hirasaki K., Liles A., Makary M.S., Molena D., Expert Panel on Interventional Radiology (2026). ACR Appropriateness Criteria Management of Chylothorax. J. Am. Coll. Radiol..

[39] Gurevich A., Hur S., Singhal S., DiBardino D., Haas A.R., Hansen-Flaschen J.H., Nadolski G., Itkin M. (2022). Nontraumatic chylothorax and chylopericardium: Diagnosis and treatment using an algorithmic approach based on novel lymphatic imaging. Ann. Am. Thorac. Soc..

[40] Fukumoto A., Terao T., Kuzume A., Tabata R., Tsushima T., Miura D., Ikeda D., Kamura Y., Narita K., Takeuchi M. (2022). Management of lymphoma-associated chylothorax by interventional radiology and chemotherapy: A report of five cases. Int. J. Hematol..

[41] Bedawi E.O., Ricciardi S., Hassan M., Gooseman M.R., Asciak R., Castro-Añón O., Armbruster K., Bonifazi M., Poole S., Harris E.K. (2023). ERS/ESTS statement on the management of pleural infection in adults. Eur. Respir. J..

[42] Rahman N.M., Maskell N.A., West A., Teoh R., Arnold A., Mackinlay C., Peckham D., Davies C.W., Ali N., Kinnear W. (2011). Intrapleural use of tissue plasminogen activator and DNase in pleural infection. N. Engl. J. Med..

[43] Akulian J., Bedawi E.O., Abbas H., Argento C., Arnold D.T., Balwan A., Batra H., Becerra J.P.U., Belanger A., Berger K. (2022). Bleeding risk with combination intrapleural fibrinolytic and enzyme therapy in pleural infection. Chest.

[44] Ferreira de Almeida M., Guimaraes F., Rodrigues I., Lima J.C., Novais Bastos H., Gomes R.E. (2026). Outcomes of intrapleural fibrinolytic and deoxyribonuclease therapy in pleural infection: A systematic review and meta-analysis. Eur. Respir. Rev..

[45] Gilbert C.R., Lee H.J., Skalski J.H., Maldonado F., Wahidi M., Choi P.J., Bessich J., Sterman D., Argento A.C., Shojaee S. (2015). The use of indwelling tunneled pleural catheters for recurrent pleural effusions in patients with hematologic malignancies: A multicenter study. Chest.

[46] Faiz S.A., Pathania P., Song J., Li L., Balachandran D.D., Ost D.E., Morice R.C., Shannon V.R., Bashoura L., Eapen G.A. (2017). Indwelling pleural catheters for patients with hematologic malignancies: A 14-year, single-center experience. Ann. Am. Thorac. Soc..

[47] Wilshire C.L., Chang S.C., Gilbert C.R., Akulian J.A., AlSarraj M.K., Asciak R., Bevill B.T., Davidson K.R., Delgado A., Grosu H.B. (2021). Association between tunneled pleural catheter use and infection in patients immunosuppressed from antineoplastic therapy: A multicenter study. Ann. Am. Thorac. Soc..

[48] Vakil E., Jimenez C.A., Faiz S.A. (2018). Pleural effusions in hematologic malignancies and their management with indwelling pleural catheters. Curr. Opin. Pulm. Med..

[49] Hughes T.P., Laneuville P., Rousselot P., Snyder D.S., Rea D., Shah N.P., Paar D., Abruzzese E., Hochhaus A., Lipton J.H. (2019). Incidence, outcomes, and risk factors of pleural effusion in patients receiving dasatinib therapy for Philadelphia chromosome-positive leukemia. Haematologica.

[50] Quintas-Cardama A., Kantarjian H., O’Brien S., Borthakur G., Bruzzi J., Munden R., Cortes J. (2007). Pleural effusion in patients with chronic myelogenous leukemia treated with dasatinib after imatinib failure. J. Clin. Oncol..

[51] Liu Y., Huang Y., Guo J., Liu X., Hu Y. (2025). Clinical characteristics and management of dasatinib-induced chylothorax: Retrospective analysis based on case reports. Front. Pharmacol..

[52] Pai T.W., Huang C.W. (2025). Low-dose dasatinib-induced chylothorax, pulmonary hypertension, and pericardial effusion in a patient with chronic myeloid leukemia: A case report and literature review. Medicine.

[53] Mirza A.-S., Kumar A., Hashmi H., Garcia F., Logothetis C.N., Darwin A., Faramand R., Reid K., Bachmeier C., Chavez J.C. (2021). Incidence and management of effusions before and after CD19-directed chimeric antigen receptor T cell therapy in large B cell lymphoma. Transplant. Cell. Ther..

[54] Goldman A., Maor E., Bomze D., Liu J.E., Herrmann J., Fein J., Steingart R.M., Mahmood S.S., Schaffer W.L., Perales M.-A. (2021). Adverse cardiovascular and pulmonary events associated with chimeric antigen receptor T-cell therapy. J. Am. Coll. Cardiol..

[55] Chen H.H., Kuo C.Y., Ho C.L., Chen Y.C. (2023). Chylothorax after chimeric antigen receptor T cell therapy for relapsed and refractory diffuse large B-cell lymphoma: A case report. Medicine.

[56] Lee D.W., Santomasso B.D., Locke F.L., Ghobadi A., Turtle C.J., Brudno J.N., Maus M.V., Park J.H., Mead E., Pavletic S. (2019). ASTCT consensus grading for cytokine release syndrome and neurologic toxicity associated with immune effector cells. Biol. Blood Marrow Transplant..

[57] Hibbert R.M., Atwell T.D., Lekah A., Patel M.D., Carter R.E., McDonald J.S., Rabatin J.T. (2013). Safety of ultrasound-guided thoracentesis in patients with abnormal preprocedural coagulation parameters. Chest.

[58] Puchalski J.T., Argento A.C., Murphy T.E., Araujo K.L., Pisani M.A. (2013). The safety of thoracentesis in patients with uncorrected bleeding risk. Ann. Am. Thorac. Soc..

[59] Fong C., Tan C.W.C., Tan D.K.Y., See K.C. (2021). Safety of thoracentesis and tube thoracostomy in patients with uncorrected coagulopathy: A systematic review and meta-analysis. Chest.

[60] Yataco A.C., Soghier I., Hébert P.C., Belley-Cote E., Disselkamp M., Flynn D., Halvorson K., Iaccarino J.M., Lim W., Lindenmeyer C.C. (2025). Transfusion of fresh frozen plasma and platelets in critically ill adults: An American College of Chest Physicians clinical practice guideline. Chest.

[61] Patel I.J., Rahim S., Davidson J.C., Hanks S.E., Tam A.L., Walker T.G., Wilkins L.R., Sarode R., Weinberg I. (2019). Society of Interventional Radiology consensus guidelines for the periprocedural management of thrombotic and bleeding risk in patients undergoing percutaneous image-guided interventions-Part II: Recommendations. J. Vasc. Interv. Radiol..

[62] Barkan D., Nusair S., Resnick I., Bitan M., Segel M., Or R., Ackerstein A., Samuel S., Breuer R., Shapira M. (2004). Tube thoracostomy during allogeneic stem cell transplantation does not carry an increased risk for infections or bleeding. Clin. Transplant..

[63] Madenci A.L., Stetson A., Weldon C.B., Lehmann L.E. (2016). Safety of peritoneal and pleural drain placement in pediatric stem cell transplant recipients with severe veno-occlusive disease. Pediatr. Transplant..

